# Crosstalk of Oncogenic Signaling Pathways during Epithelial–Mesenchymal Transition

**DOI:** 10.3389/fonc.2014.00358

**Published:** 2014-12-11

**Authors:** Stephan Lindsey, Sigrid A. Langhans

**Affiliations:** ^1^Nemours Center for Childhood Cancer Research, Alfred I. duPont Hospital for Children, Wilmington, DE, USA

**Keywords:** epithelial–mesenchymal transition, microenvironment, invasion, motility, transforming growth factor-beta

## Abstract

Epithelial–mesenchymal transition (EMT) and cell transformation have been well-documented in multiple cancer cell models and are believed to be one of the earliest events in tumor progression. Genetic and epigenetic modifications shift cells toward either end of the EMT spectrum, and can be influenced by the microenvironment surrounding a tumor. EMT and mesenchymal–epithelial transition are critical to normal function and development and an intricate network of transcription factors and transcriptional regulators tightly regulates these processes. As evidenced in normal and transformed cell lines, many signaling pathways trigger EMT during development and differentiation. The signaling pathways include those triggered by different members of the transforming growth factor superfamily, epidermal growth factor, fibroblast growth factor, hepatocyte growth factor, hypoxia-inducible factor, Wnt, Notch, and many others. Functional redundancies allow cells to undergo EMT even if these key transcriptional regulators are lacking, but these same redundancies also make these pathways particularly susceptible to gain-of-function mutations or constitutive signal activation; the “forced” transition toward either a mesenchymal or epithelial phenotype.

## Introduction

Historically, signaling pathways were studied in isolation and treated as linear entities that never interacted; however, studies in the emerging field of systems biology have provided a growing appreciation of the importance of pathway crosstalk and emphasized the complexity of signaling webs during development and tumor progression. This is especially true for the process known as epithelial–mesenchymal transition (EMT). EMT was first described in the 1980s because of its pivotal role during embryonic development and was later implicated in the physiological response to injury (1). EMT is critically involved in normal embryogenesis and development and epithelial cells have developed an intricate network of signaling pathways that include redundancies that safeguard and ensure proper functioning even in the event of a genetic lesion. However, these functional redundancies also leave the signaling network particularly susceptible to gain-of-function mutations and inappropriate signal amplification, eventually leading to tumor progression. Thus, EMT is not only a key biological process during embryonic morphogenesis but also a defining characteristic. EMT is also one of the earliest steps of solid tumor progression, associated with tumor growth, invasion, and metastasis, and contributes to the conversion of tumors from low- to high-grade malignancy (2, 3).

During EMT, epithelial cells undergo a developmental switch that results in decreased adhesion and loss of cell polarity, increased proliferation, and increased motility and invasiveness (4). Invasion is a key step to progression toward a malignant phenotype, and occurs when tumor cells translocate from the relatively constrained initial neoplastic mass into neighboring host tissues. To accomplish this, cancer cells must somehow detach from the primary tumor and migrate through surrounding tissues, opening up the opportunity to penetrate the basal-membrane surrounding a blood or lymphatic vessel, travel throughout the body via the circulatory system, and colonize distant sites where metastatic foci can be formed. Growing evidence suggests that in order for benign cancer to progress toward malignant disease tumor cells undergo EMT (5). The EMT process is associated with a number of morphological and biochemical changes where polarized and basal-membrane anchored epithelial cells acquire a mesenchymal, fibroblastoid phenotype. Morphologically, during the onset of EMT cells transform from a cuboidal epithelial-like cell to a spindle-shaped mesenchymal-like cell. These changes are associated with the down-regulation of epithelial cell surface markers and cytoskeleton components [e.g., E-cadherin, zonula occludens (ZO)-1, claudins, occludins, cytokeratins] and the up-regulation of mesenchymal markers (e.g., vimentin and α-smooth muscle actin) and extracellular matrix components (e.g., collagens and fibronectin) (6). The essential features of EMT as it relates to tumor progression are disruption of intercellular contacts and enhanced migration, the capability of matrix remodeling and tumor tissue remodeling, invasion into and migration through the extracellular matrix without the assistance of cell–cell contacts, and apoptotic resistance. Although the molecular basis of EMT have not been completely elucidated, *in vitro* and *in vivo* model systems have identified five main interconnected transduction pathways that lead to EMT and EMT-like phenotypes, many of which connect EMT to the extracellular matrix and the microenvironment surrounding tumors: tyrosine kinase receptors including the receptors for platelet-derived growth factor (PDGF), epidermal growth factor (EGF), insulin-like growth factor (IGF), hepatocyte growth factor (HGF), and fibroblast growth factor (FGF); nuclear factor kappa-light-chain-enhancer of activated B-cells (NF-κB); integrins; transforming growth factor (TGF)-β; Wnt; and many others (7). Many of these pathways share common downstream signaling effectors, highlighting the complexity of the signaling networks involved in EMT (8). In this review, we summarize some of the most prominent EMT-inducing networks and the associated molecular events leading to the transition of differentiated, polarized epithelial cells to a fibroblastic, mesenchymal cell.

## EMT-Related Signaling Networks that Regulate E-Cadherin

Most signaling pathways involved in the initiation of EMT result in the down-regulation of E-cadherin, an epithelial cell adhesion molecule that serves as a “master programmer” of EMT [recently reviewed in Ref. (9)]. A critical mediator of EMT, E-cadherin has often been described as the gatekeeper of EMT (10, 11) and in most cell types, the loss of functional E-cadherin results in loss of cell adhesion, leading to rapid cell growth and metastasis (9). In addition to its role in cell adhesion, E-cadherin is involved in transmitting signals within cells that control cell maturation, differentiation, motility, and growth. E-cadherin also acts as a tumor suppressor protein, preventing cells from growing and dividing too rapidly or in an uncontrolled way; E-cadherin down-regulation has been implicated in cell migration and invasion in murine models of mammary, prostate, and pancreatic cancer (12). Providing further correlative support for a role of E-cadherin during tumor formation, E-cadherin is inactivated in many diffuse-type cancers such as lobular breast carcinoma and gastric carcinoma, in which cells in a tumor mass lose epithelial characteristics and exhibit a highly invasive EMT-derived histological pattern. E-cadherin down-regulation occurs in solid, non-diffuse-type cancers at the tumor-stroma boundary where single EMT-derived tumor cells invade otherwise healthy tissue. In the case of single cell infiltration, E-cadherin loss and subsequent resulting EMT could be a transient, reversible process, possibly regulated by the tumor microenvironment; neoplastic cells that have undergone EMT during invasion seem to regain E-cadherin expression and their epithelial, cohesive characteristics at the secondary foci (13).

**Figure 1:**
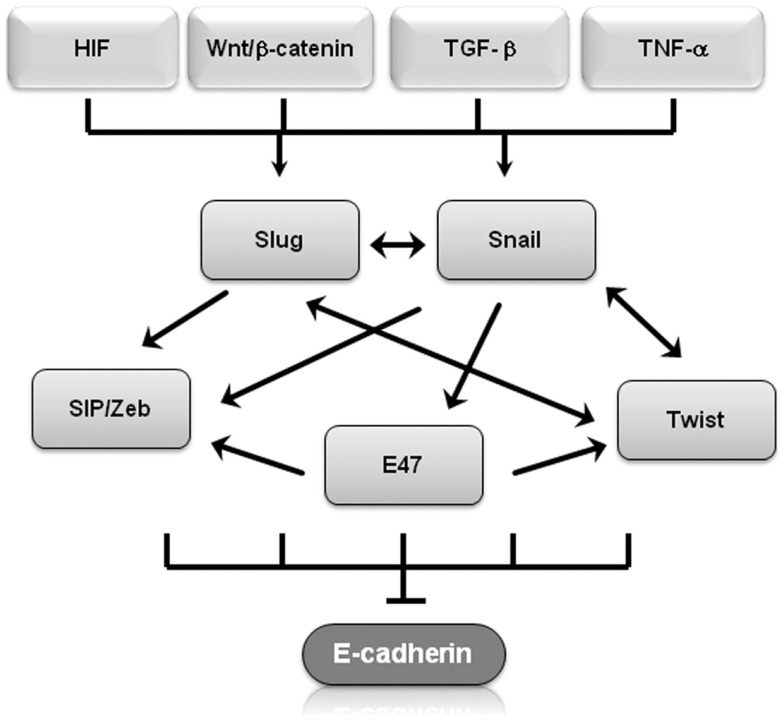
**Transcriptional regulators of EMT regulate E-cadherin**. Shown is a graphical representation of how several of transcriptional regulators of EMT regulate E-cadherin expression. Arrows represent up-regulation, T-bars represent inhibition.

Molecular events during EMT result in transcriptional regulation of the transcription factors Snail (Snail1), Slug (Snail2), zinc-finger E-box-binding homeobox (ZEB)1/2, and Twist1/2, leading to a molecular fingerprint that serves as a phenotypic marker during EMT (14). In particular, Snail, Slug, Twist, SIP1/ZEB, and E47 negatively regulate E-cadherin expression (14, 15) and display overlapping functional redundancy, in part through their common recognition of E-box sequences (Figure 1). Snail and Slug initiate EMT during development, fibrosis, and the initial invasion of cancer by repressing epithelial genes like E-cadherin by binding to E-box DNA sequences through their carboxy-terminal zinc-finger domains (16). While ZEB1/2 also binds to E-box sequences, ZEB-mediated transcriptional repression often involves the recruitment of a C-terminal-binding protein (CTBP) co-repressor (16). Twist1/2 belongs to the basic helix–loop–helix (bHLH) family of transcription factors and represses E-cadherin expression independently of Snail, probably through interactions with co-repressors (16). E47 also binds to the E-cadherin E-box, but appears to independently promote angiogenesis during tumor growth (17). Involved in most physiological EMT situations, overexpression of Snail, Slug, ZEB1/2, or Twist1/2 in epithelial cell lines typically induces EMT (18–20). These transcription factors also regulate genes other than E-cadherin. Twist and Snail have emerged as promising candidates of EMT “master genes” because they regulate genes involved in motility, proliferation, differentiation, and survival, including matrix metalloproteinases, N-cadherin, and E-cadherin in *in vitro* and *in vivo* experiments (19, 21). The signaling pathways involved in EMT should not be viewed in isolation, for evidence of interactions and crosstalk between multiple pathways exists. For example, Snail and Slug both repress E-cadherin levels and are co-expressed in various carcinomas, including breast and ovarian cancer (22). Nevertheless, by employing multiple signaling cascades, Snail and Slug could have both overlapping and simultaneously distinct roles during tumor progression, similar to what has been described for Dnmt3a/b and Vav1/2 during hematopoiesis (22–25). β-Catenin not only interacts with E-cadherin to maintain cell–cell adhesion but is also shuttled to the nucleus where Wnt serves as the transcription effector of the Wnt signaling pathway to promote proliferation and cell survival (26). This is particularly relevant during EMT because Wnt gene mutations and aberrant activation of β-catenin are considered critical events in tumor cell maintenance and growth (27). Glycogen synthase kinase (GSK)-3β-mediated stabilization of Snail is not only part of the Wnt signaling cascade but is also required in colorectal cancer cells for EMT induced by the pro-inflammatory cytokine tumor necrosis factor (TNF)-α (28). Slug was identified as a downstream Wnt signaling pathway effector in a basal-like carcinoma model that also linked the Wnt pathway to tumor proliferation and self-renewal (29). These finding suggest that Slug and Wnt play important roles in maintaining the stemness of human mammary tumor cells.

## EMT and Circulating Cancer Cells

It is believed that some cells slough off the outer edges of a tumor as it proliferates and are swept away by the bloodstream or lymphatic system (30). These rare so-called circulating tumor cells (CTCs) from a primary tumor have the capacity to be shed into the vasculature, where they circulate throughout the bloodstream and eventually find a suitable location to colonize and form distant metastases in new tissues (30). One of the key features of the CTC theory of metastasis is that these cells are able to become quiescent until microenvironmental conditions favor growth. Interestingly, two EMT-inducers, Snail and Twist, are upregulated in CTCs and allow cancer cell populations to revert to a stem-cell-like quiescent state (31). Further supporting a link between EMT and CTCs, more than 80% of CTCs collected based on the expression of an epithelial marker (EpCAM) also expressed a mixture of epithelial and mesenchymal markers, suggesting that these CTC were currently transitioning along the EMT axis (32). Indeed, these cells expressed many molecular markers only seen in cells undergoing EMT, including epithelial proteins such as cytokeratin and E-cadherin; mesenchymal proteins including vimentin, N-cadherin, and O-cadherin; and the stem cell marker CD133 (32). Additionally, CTC-like cells increased after immortalized human mammary epithelial cells were transfected with Ras to initiate EMT (33, 34). Further implicating EMT in CTC production, CTCs obtained from metastatic breast cancer patients express a much higher rate of Twist and vimentin, two markers of EMT, than patients with early breast cancer (35). Many of the same microenvironmental cues that regulate EMT also seem to be upregulated in CTCs, especially hypoxia and inflammatory mediators such as NFκB and TGFβ (36). Inflammatory signaling mediators emanating from the microenvironment also play a critical role in the maintenance of CTCs. The receptor for stromal-derived-factor-1 (SDF-1), C–X–C chemokine receptor 4 (CXCR4) enhances the survival of CTCs as they circulate throughout the body (37). Microenvironmental signals also serve as cues to tell CTCs to leave the circulation and to colonize distant organs. In breast cancer, bone marrow cells secrete parathyroid hormone-related protein (PTHRP), TNF-α, interleukin 6 (IL-6), and/or IL-11 stimulate the release of the receptor activator of NF-κB ligand (RANKL) from osteoblasts and suppress the release of the RANKL antagonist osteoprotegerin, allowing for breast tumor CTCs to colonize the bone marrow (37). TNF-related apoptosis-inducing ligand (TRAIL) has also been recently shown to contribute to EMT by miR-221-induced suppression of phosphatase and tensin homolog (PTEN) (38). Similarly, interactions between endothelial selectins and selectin ligands expressed on CTCs, up-regulation of adhesion molecules, and interactions between adherent neutrophils within inflamed sinusoids and CTCs contribute metastatic colonization of the liver (39). Together, these examples paint a complex picture of signaling crosstalk that intersect at the CTC and play a critical role in tumor progression.

## MicroRNA Regulation of EMT

MicroRNAs (miRNAs) are small non-coding RNA molecules that play key roles in the regulation of transcriptional and post-transcriptional gene expression (40). In addition to their important roles in healthy individuals, miRNAs are important players during EMT and are differentially expressed in a broad range of cancers (41). Because a single miRNA can target several messenger RNAs, dysregulation of miRNAs can influence multiple signaling pathways leading to tumor formation and metastasis. For example, miR-138 controls EMT by targeting at least three genes: vimentin, ZEB2, and enhancer of zeste homolog EZH2 (42). Similarly, the miR-200 micro RNA family targets at least two transcriptional repressors of E-cadherin, ZEB1 and ZEB2; altering miR-200 in transformed cell lines induced changes consistent with either inducing EMT or the reverse process, mesenchymal–epithelial transition (MET) (43). Reduced expression of miR-30a promotes TGF-β-induced EMT by targeting SNAI1 (44). In addition to the control of transcription factors, miRNA also affects multiple aspects of the EMT process, including increased motility and invasiveness, cell adhesion, disassembly of epithelial cell junctions, and destabilization of tight junctions (45).

## Genetic Lesions and Epigenetic Modifications in EMT

Many signaling pathways associated with EMT result in increased cellular proliferation and create feedback loops, resulting in a perpetual proliferative state during the initial stages of EMT. In normal, healthy cells, genomic integrity during cell division is ensured by DNA repair and cell cycle checkpoints that respond to DNA damage by inhibiting critical cell cycle events (46). However, the increased proliferation rate in cells undergoing EMT provides tumor cells an opportunity to proceed through mitosis without high-fidelity proofreading and/or repair, consequently, resulting in the potential of increased mutation rates (47). However, increased proliferation during EMT is not sufficient for tumor development on its own and additional genetic lesions are required to move past the initial cellular dysplasia toward a malignant tumor. Consistent with this model, the carcinogenic potential of estradiol, for example, is thought to be mediated by a combination of proliferation and increased mutation rate [reviewed in Ref. (48, 49)].

Epigenetic deregulation of gene expression is involved in the initiation and progression of multiple cancers and an important initiator of EMT. Similar to its role in differentiation, development, and malignant transformation, epigenetic reprograming during EMT is largely mediated by chromatin remodeling (50). DNA methylation patterns are preserved during EMT and sustained EMT activation leads to epigenetic alterations, inducing heritable changes that maintain the mesenchymal phenotype even after EMT-initiating signals are removed. Epigenetic modifications, especially histone and DNA methylation, are critical to gene regulation and establish patterns of gene repression during development and EMT (51). Snail represses E-cadherin expression by forming a co-repressor complex with histone deacetylase HDAC1 and HDAC2, resulting in E-cadherin silencing and *in vivo* pancreatic cancer progression (52). Overexpression of the histone methyltransferase MMSET (multiple myeloma SET domain) in prostate cancer influences histone 3 lysine 36 dimethylation (H3K36me2) and lysine 27 tri-methylation (H3K27me3). MMSET overexpression in immortalized prostatic epithelial cells leads to increased migration, increased invasion, morphological changes, and altered gene expression consistent with transition from an epithelial cell-like state to a mesenchymal cell-like state (53). Mediated by the ability of MMSET-mediated activation of *TWIST1*, a gene implicated in tumor-associated EMT and invasion (19), these data suggest that deregulated MMSET results in aberrant epigenetic gene regulation, leading to tumor progression and metastasis. Genome-wide histone maps focusing on H3 lysine 4 and lysine 27 tri-methylation (H3K4me3 and H3K27me3) identified differentially expressed genes in embryonic stem cells (54–57), hematopoietic stem cells/progenitor cells (58), T cells (59), and in prostate cancer cells (60). Although DNA methylation has been implicated in the transition from EMT to MET, reversible histone modifications are the predominant factors in reactivation of E-cadherin expression during the transition from EMT to MET (61).

Genome-scale mapping revealed that most chromatin changes are heterochromatin K9-modifications, suggesting that EMT is characterized by the epigenetic reprograming of specific, large chromatin domains across the genome (50). Similarly, clustered chromatin profiles using combinatorial patterns of posttranslational histone modifications and covalent changes to genomic DNA discovered a distinct chromatin signature among genes in well-established EMT pathways including the epidermal growth factor receptor (EGFR), suggesting that chromatin remodeling of EGFR plays an important role in EMT (62). Acetylation, regulated mainly through HDACs also affects EGFR expression and downstream signaling. HDAC6 up-regulation slows EGFR endocytic trafficking from early endosomes to late endosomes in renal epithelial cells and HDAC6 inhibition results in decreased phosphorylation of extracellular signal-regulated kinase (ERK) 1/2, a downstream target of EGFR (63). Future experiments should determine if these findings are common to EMT and determine if similar epigenetic reprograming occurs in other physiological contexts. Aside from this role in epigenetic reprograming, crosstalk between the more traditionally known EGFR signaling cascade and components of other signaling pathways frequently leads to abnormal activation of pro-proliferative and anti-apoptotic pathways. The most common signaling cascades activated by EGFR are the phosphatidylinositol-3-kinase (PI3K)/Akt, Ras/Raf/Mek/extracellular signal-regulated kinase, and the Jak/Stat pathways (64) that both contribute to the development of malignancies by impacting cell cycle progression, inhibition of apoptosis, angiogenesis, tumor cell motility, and metastases (65). Crosstalk between EGFR and other signaling pathways impact cancer treatment as well as the initiation of EMT. For example, one well-known mechanism of resistance to the selective EGFR inhibitor gefitinib/erlotinib is HGF receptor tyrosine kinase gene amplification. HGF receptor tyrosine kinase gene amplification bypasses normal EGFR signaling to instead activate AKT through HER3-mediated activation of PI3K in the presence of EGFR tyrosine kinase inhibitors (66).

## Crosstalk between TGFβ and Other Signaling Pathways Mediating EMT

**Figure 2:**
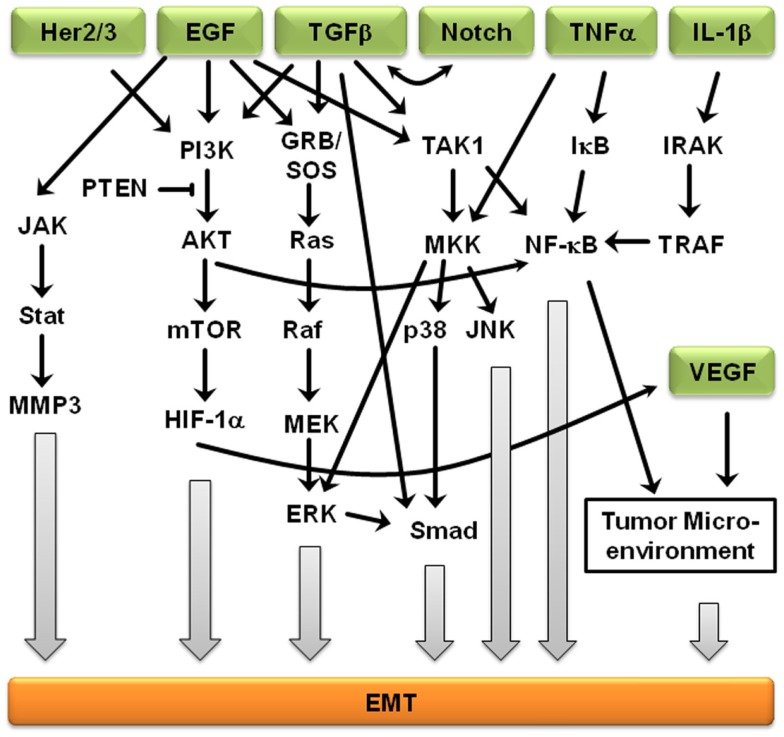
**Crosstalk between EMT-inducing signaling pathways**. Representation of some of the points of intersection between various EMT-inducing signaling pathways. For clarity, branches that did not result in crosstalk are not shown. Arrows represent up-regulation.

Signaling pathways are not independent from each other, but rather interact to form complex signaling networks; the TGFβ signaling pathway is no exception. Most likely, due to its involvement during many cellular processes including proliferation, differentiation, apoptosis, and cellular homeostasis, the TGFβ pathway interacts with many other signaling pathways during EMT (Figure 2). One mechanism by which TGFβ initiates EMT is by removing β-catenin from adherens junctions in a process that involves TGFβ-dependent PTEN dissociation from β-catenin and Akt activation (67). Depending on the context, Notch can either synergize with TGFβ/bone morphogenetic protein (BMP) signals to induce target genes or inhibit TGFβ/BMP signaling (68–71). In the presence of other growth factors, TGFβ/BMP signaling generally stimulates migration and blocks endothelial cell proliferation, but Notch signaling inhibits the migratory affect of BMP (72). Stimulation of endothelial cells with BMP alone promoted cell migration, but in the presence of Notch signaling, cell migration was inhibited (72). Interestingly, the dominance of Notch signaling over BMP signaling was cell–cell contact-dependent, suggesting that endothelial cells not in contact with surrounding cells are stimulated by TGFβ to migrate until new cell–cell contacts are established, at which point Notch induces gene expression changes and arrests further migration (72). Similarly, Notch signaling is necessary for growth arrest by TGFβ in epithelium; over 30% of TGFβ-induced epithelial genes require Notch signaling for full expression (68). TGFβ leads to increased jagged-1 expression and siRNA-mediated knockdown of jagged-1 leads to reduced TGFβ-induced p21 expression, rescuing TGFβ-inhibited proliferation (68). Therefore, TGFβ induces both c-myc, which stimulates cell cycle progression, and jagged-1, which blocks cell cycle progression through stimulation of Notch and induction of p21. Jagged-1 induction is rapid and transient, so a balance between TGFβ/Notch-induced p21 and TGFβ/Smad-induced c-myc may act as a switch to regulate cell proliferation (72).

Further supporting the importance of crosstalk during EMT, Erk, c-Jun N-terminal kinase (JNK), and p38 indirectly regulate TGFβ signaling, but TGFβ treatment leads to activation of Erk1/2 and mitogen-activated protein kinase (MAPK) signaling (73). Smad-dependent signaling and MAPK-mediated Erk1/2 activation is believed to result in cross-talk between the TGFβ and EGF signaling pathways (74). The MAPK/Erk signaling pathway also mediates Smad2/3 phosphorylation and nuclear exclusion, which is believed to be important for the attenuation of TGFβ-induced nuclear translocation of MAPK (74). MAPK signaling also results in Smad1/5 phosphorylation, leading to an inability to translocate specific Smads into the nucleus (74). Erk-mediated Smad1 phosphorylation creates a docking site for the Smad1/5-specific E3 ubiquitin ligase, Smurf1. Smurf1 binding results in Smad ubiquitination and eventual degradation and blocks Smad interactions with the nuclear pore complex. MAPKs also regulate the protein stability of Smad4 and the inhibitory Smad7 (74), suggesting that Smad3 is indispensable to the mediation of the pro-apoptotic effects of TGFβ, Smad3, but not the closely related Smad2, is the primary target of PI3K/Akt-mediated inhibition (74). In addition, TGFβ regulates Akt activity and phosphatase and tensin homolog (PTEN) function during EMT initiation. In addition to activating the MAPK and PI3K/Akt pathways, ErbB signaling interacts with TGFβ/Smad during development and breast cancer progression (75). The PI3K/Akt pathway is also subjected to TGFβ regulation. Akt activity increases in response to TGFβ treatment, which seems to be required for a variety of TGFβ-induced activities, such as cell migration of HER2-expressing breast cancer cells, EMT of normal mammary epithelial cells, cell survival of mouse hippocampal neurons and mesenchymal cells, as well as growth stimulation of certain fibroblasts (74, 75). EGFR and IL-6R signaling cross-talk through JAK2/STAT3 to mediate EMT in ovarian carcinomas; activated STAT3 in high-grade ovarian carcinomas may occur directly through activation of EGFR/IL-6R or indirectly through induction of IL-6R signaling (76). Another ligand of EGFR, TNF-α, also induces EMT through NF-κB-mediated transcriptional up-regulation of Twist1 (77). In breast cancer-related EMT, HER2/Ras antagonizes TGFβ-induced apoptosis and cell cycle arrest while simultaneously enhancing the pro-migratory and pro-invasive functions of TGFβ (78). TGFβ transcriptionally downregulates PTEN in Smad4 null pancreatic cancer cells and relies on the function of the Ras/MAPK pathway (73–75). EMT-related crosstalk is also clinically relevant; pharmacological blockade of IGF-1R fully prevented TGFβ’s ability to activate an EMT protein signature (79).

## Involvement of the Microenvironment during EMT

**Figure 3:**
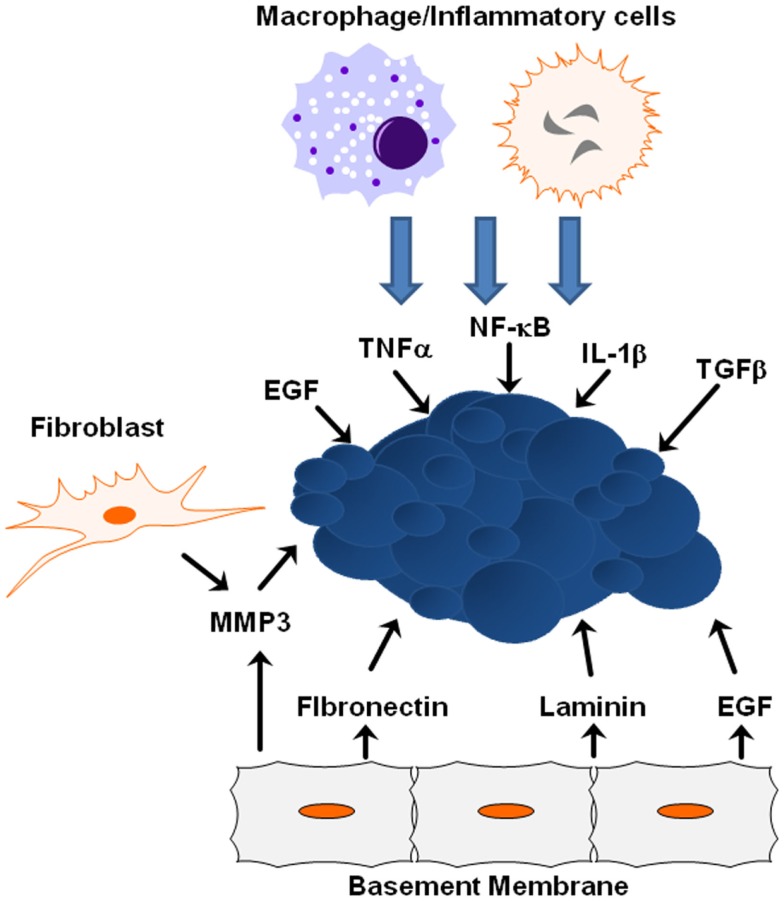
**ECM/growth factor involvement in EMT**. Shown are several molecular mechanisms by which the cells within the tumor microenvironment can influence tumor progression and EMT initiation.

The tumor microenvironment plays a crucial role in tumor progression and metastasis, and as tumors develop, the integrity of the surrounding basement membrane plays a critical role in invasion and metastasis. The tumor microenvironment is composed of inflammatory and immune cells, physical interactions with neighboring cells, oxygen and nutritional gradients, stromal extracellular matrix, and soluble factors. Cells neighboring the developing tumor secrete growth factors and inhibitory molecules that regulate tumor proliferation and apoptosis, while tumor cells simultaneously secrete factors to neighboring cells that regulate adhesion. The temporal–spatial changes within the microenvironment surrounding tumors and the interaction between tumor cells and their microenvironment are crucial to tumor initiation and development, and are especially critical to cancer cell quiescence, tumor progression, invasion, tumor metastasis, and drug resistance (Figure 3) (80).

Many signals received from the tumor microenvironment can initiate EMT including TGFβ, hypoxia-inducible factor (HIF)-1α, EGF, WNTs, and Notch (21). Various signals trigger expression of these transcription factors including heterotypic interactions with neighboring cancer cells and interactions with adjacent tumor-associated stromal cells. Epithelial–mesenchymal interactions within the tumor microenvironment integrate several important signaling molecules that are critical for tumor growth and metastasis, including integrins, cytokines, and growth factors (81). Crosstalk between the TGFβ and HER2/Ras/MAPK signaling pathways often leads to secretion of additional growth factors and cytokines, including TGFβ itself, which in turn promotes EMT and cell invasion, whereas JNK kinases negatively regulate the autocrine expression of TGFβ1 (73, 75). ErbB receptors and their ligands are also involved in cross-talk between cancer cells and the tumor microenvironment. EGFR is activated in tumor-associated endothelial cells, but not in endothelial cells within uninvolved organ regions, suggesting that EGFR activation and expression is partially determined by the tumor microenvironment (82). As time goes on, the importance of the microenvironment to pathogenesis is becoming clearer, from the role the ECM and matrix rigidity plays in determining polarity, to the extracellular metabolism of growth factors and matrix molecules during cancer progression and metastasis.

Extracellular matrix proteins and physical properties within the microenvironment can lead to tumor progression by activating EMT-inducing pathways within tumors. One key extracellular matrix protein is matrix metalloproteinase-3 (MMP3), a matrix-degrading enzyme secreted by stromal fibroblasts known to induce *in vitro* and *in vivo* EMT in mammary epithelial cells (83). When tumor cells are exposed to MMP3, transcription of a splice variant of Rac1 called Rac1b increases stimulating the production of reactive oxygen species and expression of Snail1 (84). EGFR activation in human carcinoma cell lines increases MMP-9 activity and is associated with increased *in vitro* cell invasion (85). Synthetic low-molecular weight or endogenous MMP inhibitors or an anti-catalytic MMP-9 antibody blocked increased invasive activity after EGF-mediated induction, indicating EGFR activation results in enhanced MMP-9 expression and may facilitate the removal of extracellular matrix barriers to tumor invasion. Additional proteins within the basement membrane influence EMT induction from ectopic exposure of MMP3. For example, the laminin suppresses EMT in MMP3-treated cells, while fibronectin promotes EMT due to interactions with specific integrin receptors (7). During this process, α6-integrin sequesters Rac1b from the basement membrane and is required for inhibition of EMT by laminin; α5-integrin maintains Rac1b at the membrane and is required for the promotion of EMT by fibronectin (7). Additionally, matrix rigidity may also play an important role during EMT. The microenvironmental stiffness surrounding cells impacts differentiation and response to external molecular cues, while epithelial cells treated with MMP3 undergo EMT when cultured on plastic or glass, cells cultured on soft matrices do not undergo EMT in response to treatment with MMP3 (7).

## Involvement of Inflammatory Signaling in EMT

The microenvironment surrounding a tumor is often dominated by inflammatory cytokines that promote tumor initiation by leading to increased angiogenesis, tumor growth, and tumor progression (86). Tumor-associated macrophages secrete EGF to neighboring cancer cells, which in turn stimulate macrophages to facilitate intravasation and metastatic dissemination of the cancer cells (87, 88). Together, these findings substantiate a role of EGF-mediated signaling not only in EMT and proliferative signaling itself but also in the cross-talk between tumor cells and the microenvironment. The tumor microenvironment is largely orchestrated by inflammatory cells, which facilitate extracellular matrix breakdown, angiogenesis, and tissue remodeling, thus, promoting tumor cell motility (89). Inflammatory cells play a major role in secreting activating factors that lead to NF-κB activation; NF-κB is a key regulator of the inflammatory response shown to regulate Slug and Snail (90). TGF-β activity is deregulated during malignant cancer progression, and plays an important role in EMT (91). Similarly, both TNFα and interleukin-1β (IL-1β) are expressed at low levels in normal breast epithelial cells, but are upregulated in the majority of breast cancer patients, with pronounced expression of both cytokines in over 80% of patients who experience breast tumor relapse (92). *In vivo* murine breast models suggest increased expression and activity of TNFα results in many cancer-promoting functions and that inhibition of TNFα expression leads to reduced breast cancer malignancy (93). Chronic TNFα expression in the tumor microenvironment is correlated with a more aggressive tumor phenotype (93). IL-1β upregulates a variety of processes that contribute to higher angiogenesis, tumor growth, and tumor progression and is considered a strong and causative pro-inflammatory factor whose expression is associated with advanced cancer (94). TNFα impacts cell morphology and may cooperate with TGFβ to lead to EMT in non-transformed breast epithelial cells (95). Sustained co-expression of TNFα and IL-1β acts through the complex regulatory processes of the EMT activators Zeb1, Snail, and Twist to result in morphologic changes including cell spreading, protrusion formation, decreased E-cadherin expression, and increased expression of vimentin, all consistent with EMT (96).

## Hypoxia and EMT

When microenvironmental cues are favorable for growth, rapid cell growth with a tumor results in local hypoxia and nutrient deficits, regardless of the oxygen tension surrounding the tumor (97). Therefore, sustained tumor growth requires increased local vasculature to provide oxygen and metabolites to feed the growing tumor (98) and the nutritionally impoverished and hypoxic environment within tumors results in local changes in hypoxia-related gene expression, contributing to tumor heterogeneity (99). Tumor cells adjust to hypoxia and lack of nutrients not only by activating specific pathways associated with angiogenesis but also associated with hypermetabolism, glycolysis, and resistance to acidosis-induced toxicity (100). Hypoxia genes, especially HIF-1α, are frequently upregulated within many solid tumors and promote tumor progression (101, 102). HIF-1α induces EMT and self-renewal of cancer stem cells, and facilitates metastasis; knockdown of HIF-1α inhibits or even reverses the EMT-like phenotype (103, 104). Hypoxia-induced EMT is mediated by HIF-1α via up-regulation of transcription effectors such as Snail, Twist, and ZEB1/2 and results in the suppression of E-cadherin expression (105–107). Several additional signaling pathways that are critical for embryonic development including Notch, Wnt, and TGFβ are also involved in hypoxia-induced EMT. Demonstrating a complex integration of hypoxic signals into EMT, Notch signaling directly upregulates Snail, and potentiates HIF-1α recruitment to the lysyl oxidase promoter, resulting in stabilization of Snail, increased cell motility, and increased invasiveness (108). Similarly, Wnt/β-catenin signaling enhances hypoxia-induced EMT by increasing the EMT-associated activity of HIF-1α and preventing cell death (109). Hypoxia also inhibits Wnt signaling by interfering with β-catenin acetylation (110), blocking secretion of Wnt (111), and activating Siah-1 in a p53-dependent manner (112).

Depending on cell type, Wnt/β-catenin signaling also enhances hypoxia-induced EMT by increasing the EMT-associated activity of HIF-1α and preventing tumor cell death (109). HIF-1α also competes with T-cell factor-4 (TCF-4) to bind β-catenin and form a HIF-1α/β-catenin complex, accompanied by increased HIF-1α transcriptional activity in colorectal tumors (113). TGFβ signaling also integrates hypoxia-related cues, for TGFβ/Smad3 inhibit vascular smooth muscle cell apoptosis through an autocrine signaling mechanism involving VEGF (114). Adding further complexity to the impact of TGFβ signaling, TGFβ not only activates the Notch signaling pathway but Notch signaling also activates TGFβ in rat mesangial cells under high-glucose conditions (68, 115). It is important to note that this example also highlights how signals from the microenvironment can influence signaling outcomes.

## Conclusion

Epithelial–mesenchymal transition is a key physiological process during normal development and regulated by an intricate network of signaling pathways that allows for the integration of signaling cues during embryonic morphogenesis. While these signaling networks allow for the precise control required for a major switch from a differentiated epithelial cell into mesenchymal cell, it also opens up the possibility of deregulation on multiple levels during pathological processes such as cancer and fibrosis. Owing to the complex interactions between these signaling pathways, activating mutations in signaling molecules can be amplified. Many of these potentially deregulated pathways converge on a few master regulatory molecules or parallel pathways can induce changes on various levels. Thus, it is plausible that EMT contributes to cancer progression in various ways, including tumor growth, invasion, and metastasis. Moreover, depending on the nature of the genetic lesions, EMT can become a very individualized process, adding to the complexity of cancer, while also opening up the possibility of personalized medicine. Thus, our improved understanding of EMT signaling networks and their association with therapeutic resistance is imperative for future development of novel anti-tumor drug and treatment strategies, especially in high-grade tumors and tumors that have developed therapeutic resistance.

## Conflict of Interest Statement

The authors declare that the research was conducted in the absence of any commercial or financial relationships that could be construed as a potential conflict of interest.

## Abbreviations

bHLH, basic helix–loop–helix; BMP, bone morphogenetic protein; CTBP, C-terminal binding protein; CTC, circulating tumor cell; CXCR4, C–X–C, chemokine receptor 4; EGFR, epidermal growth factor receptor; EMT, epithelial–mesenchymal transition; ERK, extracellular signal-regulated kinase; FGF, fibroblast growth factor; GRB2, growth factor receptor-bound protein 2; GSK, glycogen synthase kinase; HDAC, histone deacetylase; HGF, hepatocyte growth factor; HIF, hypoxia-inducible factor; IGF, insulin-like growth factor; IκB, nuclear factor of kappa light polypeptide gene enhancer in B-cells inhibitor, alpha; IL-6 interleukin 6; IRAK, interleukin-1 receptor-associated kinase; JNK, c-Jun N-terminal kinase; MAPK, mitogen-activated protein kinase; MET, mesenchymal–epithelial transition; MMP3, matrix metalloproteinase-3; MMSET, multiple myeloma SET domain; NF-κB, nuclear factor kappa-light-chain-enhancer of activated B-cells; PDGF, platelet-derived growth factor; PI3K, phosphatidylinositol-3-kinase; PTEN, phosphatase and tensin homolog; PTHRP, parathyroid hormone-related protein; RANKL, receptor activator of NF-κB ligand; SDF-1, stromal-derived-factor-1; SOS, son of sevenless; TGF, transforming growth factor; TNF, tumor necrosis factor; TRAIL, tumor necrosis factor-related apoptosis-inducing ligand; TRAF, TNF receptor-associated factor; ZEB, zinc-finger E-box-binding homeobox; ZO, zonula occludens.
